# Endovascular Management of an Infrarenal Abdominal Aortic Aneurysm With Severe Tortuosity of the Left Common Iliac Artery: A Case Report

**DOI:** 10.7759/cureus.94087

**Published:** 2025-10-07

**Authors:** Fikri Taufiq, Saskia D Handari, Yan E Sembiring

**Affiliations:** 1 Department of Cardiology and Vascular Medicine, Faculty of Medicine Universitas Brawijaya, Malang, IDN; 2 Department of Cardiology and Vascular Medicine, Saiful Anwar Hospital, Malang, IDN; 3 Department of Cardiology and Vascular Medicine, Faculty of Medicine Universitas Brawijaya, Surabaya, IDN; 4 Cardiac Imaging, Siloam Hospital Surabaya, Surabaya, IDN; 5 Department of Cardiovascular Surgery, Premier Hospital Surabaya, Surabaya, IDN

**Keywords:** abdominal aortic aneurysm, aorta-uni-iliac stent-graft, common iliac artery, crossing techniques, endovascular aneurysm repair

## Abstract

Abdominal aortic aneurysms (AAA) require careful management. Endovascular aneurysm repair (EVAR) is a less invasive alternative to open surgery, but complex aneurysms involving challenging iliac artery anatomy may require advanced procedures. A 72-year-old man presented with a symptomatic infrarenal AAA and a severe tortuosity of the left common iliac artery. Due to this unfavorable anatomy, a complex EVAR approach was undertaken. This involved occlusion of the left common iliac artery using an Amplatzer Vascular Plug II (Abbott Laboratories, Abbott Park, IL), followed by deployment of an aorto-uni-iliac EVAR stent graft via the right femoral artery. To maintain left lower extremity perfusion, a femoral-femoral crossover bypass graft was created. The patient recovered without complications. This case demonstrates the feasibility and effectiveness of complex EVAR techniques in managing challenging aortic aneurysms, emphasizing the need for careful patient selection, meticulous technique, and close follow-up to ensure successful outcomes.

## Introduction

An abdominal aortic aneurysm (AAA) is a serious condition requiring careful management. Rupture of an AAA carries a high mortality rate, making timely intervention critical [1]. EVAR has emerged as a less invasive strategy than open surgical repair, offering lower mortality and morbidity rates [2]. However, anatomical constraints, such as challenging iliac artery anatomy, can complicate EVAR procedures. Careful preoperative planning, including appropriate stent graft sizing and placement, is crucial to avoid complications such as endoleaks. This report describes the case of a 72-year-old man with an infrarenal AAA and severe tortuosity of the left common iliac artery, characterized by an angulation exceeding 60 degrees, necessitating a complex EVAR approach involving left common iliac artery occlusion, EVAR, and a femoral-femoral crossover bypass. The patient has comorbidities, including hypertension, contributing to perioperative risk. His overall physical status was classified as ASA II, reflecting a patient with severe systemic disease but without an imminent threat to life.

## Case presentation

A 72-year-old man presented with chest and dull abdominal discomfort for three months, with a pain scale score of 3 out of 10, despite receiving optimal medication to control hypertension and chronic coronary syndrome. The patient reported a history of hypertension, but denied any history of peripheral arterial disease. An electrocardiogram showed sinus rhythm with a normal ST-T segment. A chest X-ray revealed cardiomegaly. Laboratory results showed normal hemoglobin (13.9 mg/dL) and normal renal function (ureum 31.2 mg/dL, creatinine 1.11 mg/dL, and eGFR 87 ml/min/1.73 m^2^).

A computed tomography scan showed a coronary artery calcium score of 519, and a CT coronary angiogram showed non-significant stenosis in the proximal-mid left anterior descending artery, proximal left circumflex artery, and proximal-mid right coronary artery. Due to the disparity between the patient's clinical history and the laboratory findings, further examination of the abdominal aorta was performed. An abdominal ultrasound demonstrated an abdominal aortic diameter of 50.8 mm.

**Figure 1:**
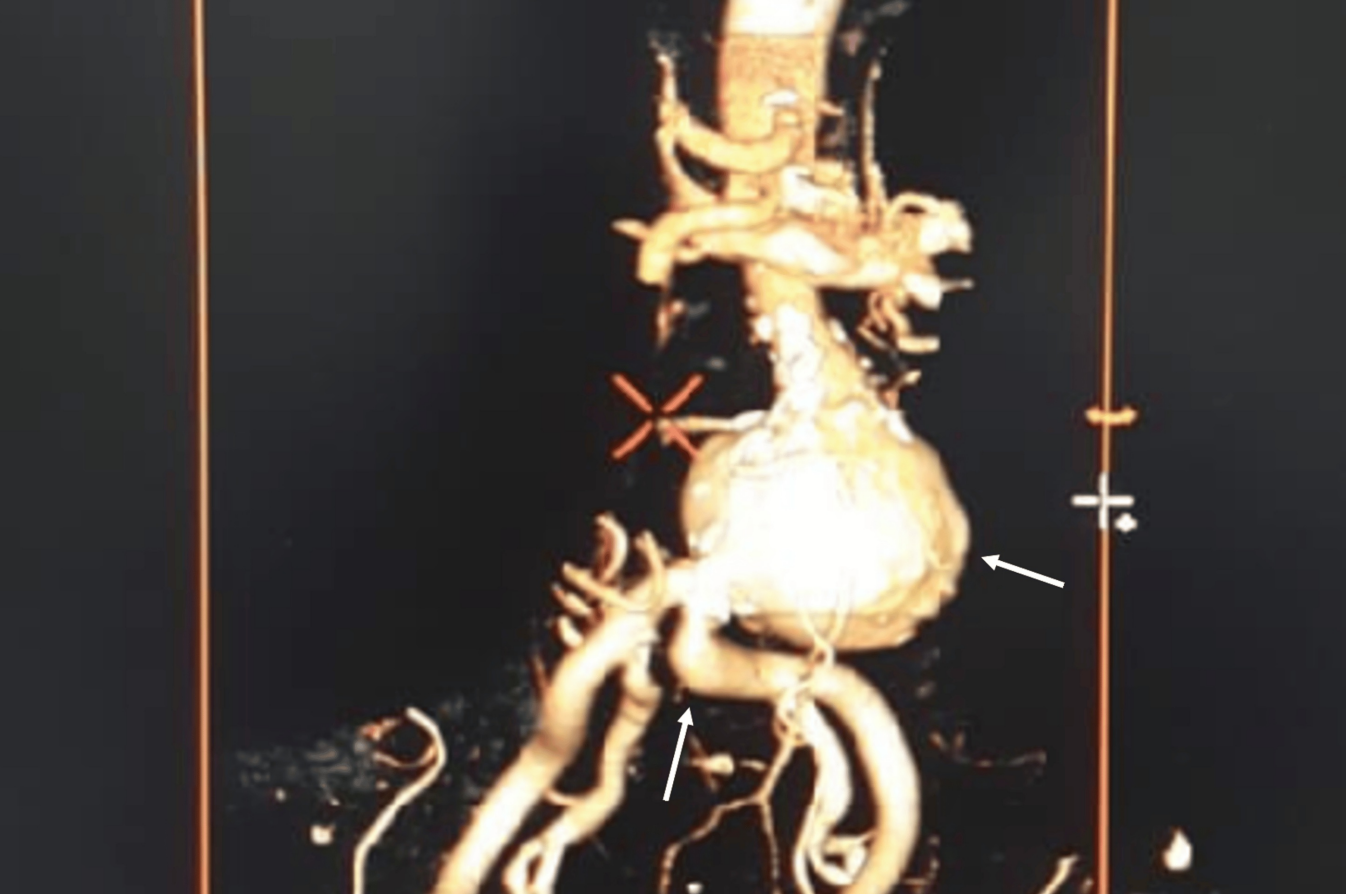
CT aortoiliac angiography revealed an infrarenal abdominal aortic aneurysm with severe tortuosity of the left common iliac artery.

Cardiovascular computed tomography (CCT) revealed the following: Proximal aneurysm located 50 mm inferior to the right renal artery and 40 mm inferior to the left renal artery, aneurysm length of 56 mm with a maximal diameter of 63 mm, no signs of dissection or contrast extravasation, normal celiac trunk and superior mesenteric artery and their branches, root of the inferior mesenteric artery and right and left third lumbar arteries originating from the aneurysm segment, and severe tortuosity of left common iliac artery, characterized by an angulation exceeding 60 degrees (Figure 1).

**Figure 2:**
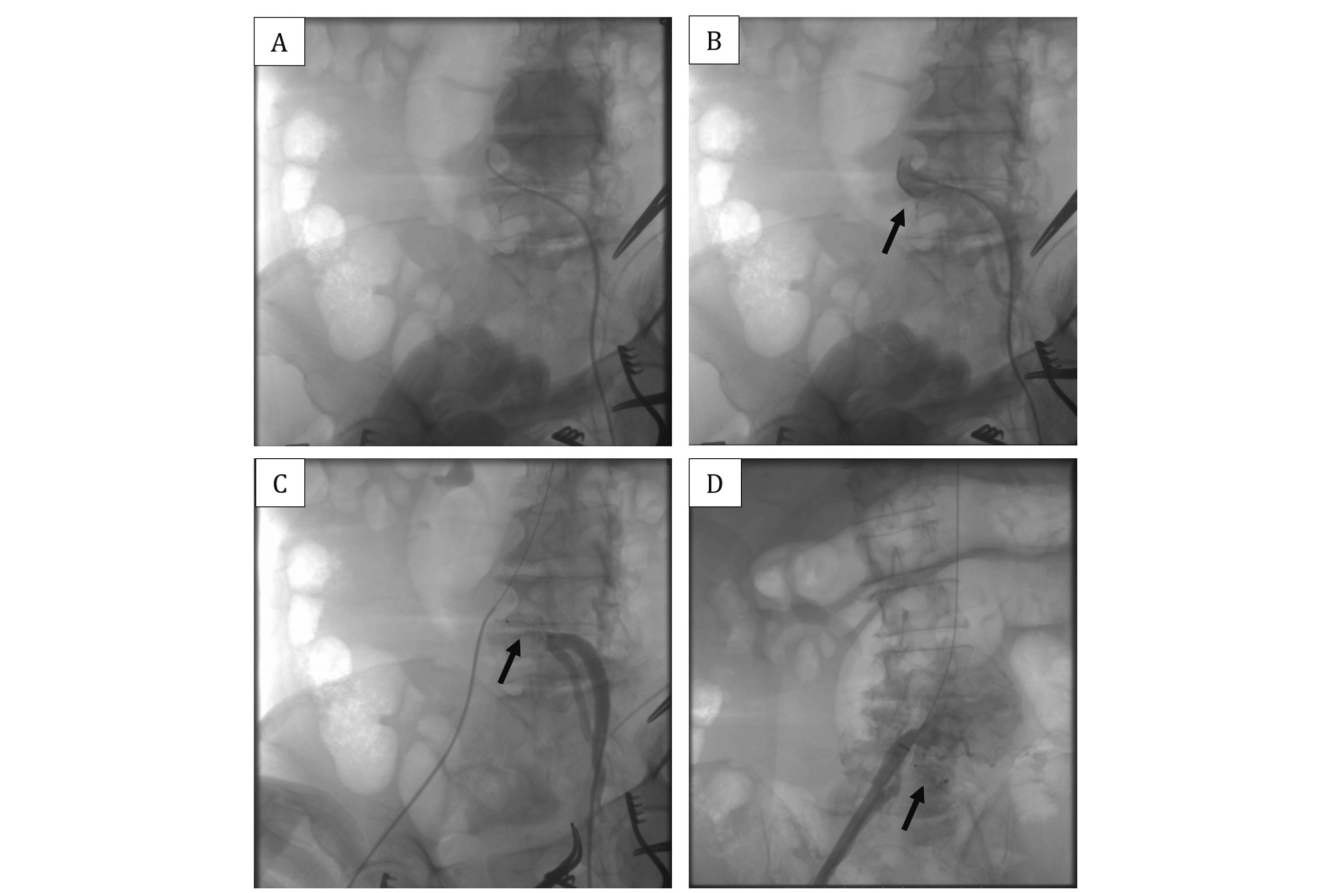
Pre-intervention angiography demonstrates severe tortuosity of the left common iliac artery (A and B). An Amplatzer™ Vascular Plug II 14/10 (Abbott) was deployed to achieve occlusion of the left common iliac artery and post-intervention angiography confirms successful occlusion (C and D).

**Figure 3:**
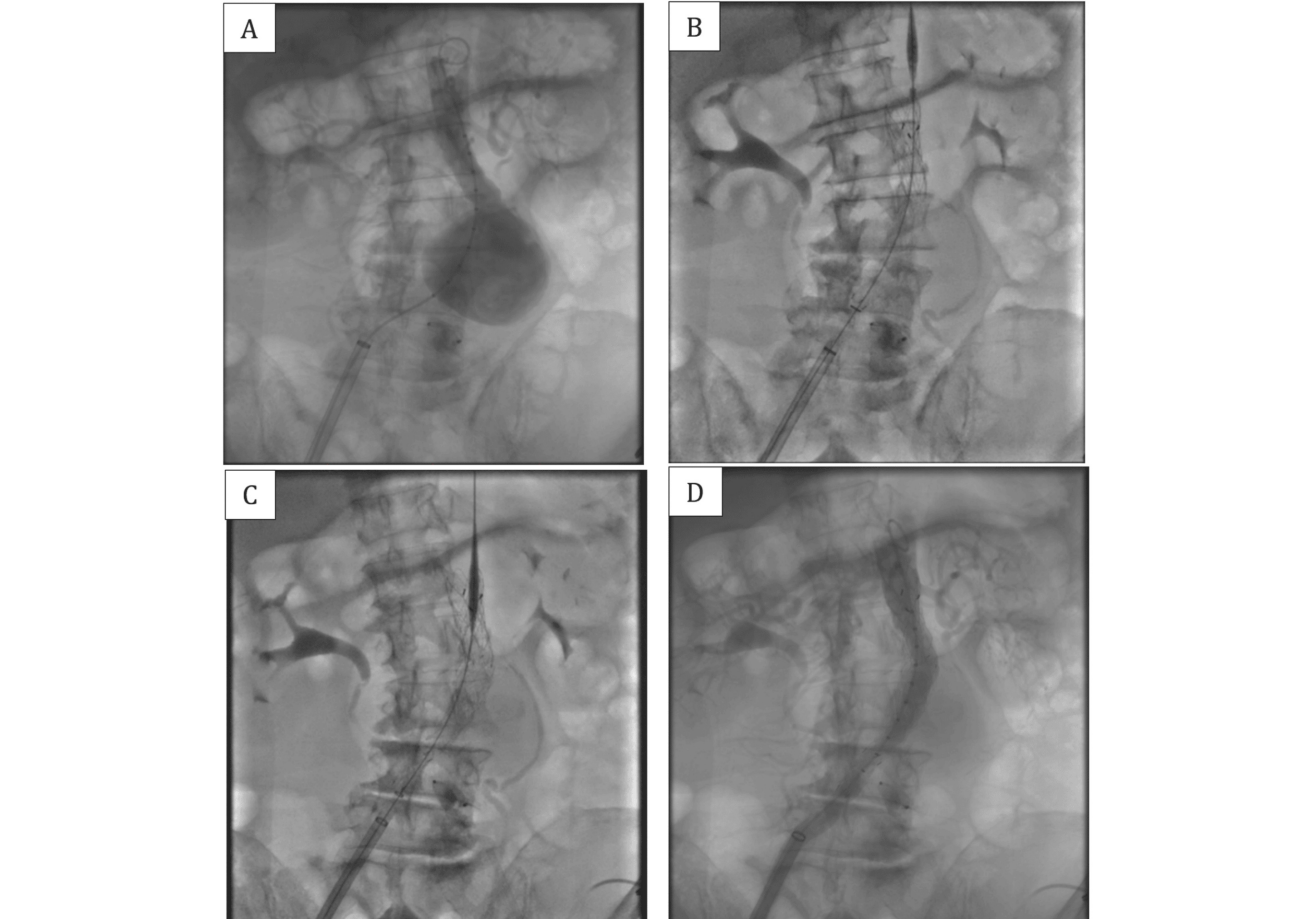
Angiography demonstrated contrast filling the aneurysm before the EVAR procedure (A). An aorto-uni-iliac endograft (SEAL NOVUS Body Stent Graft 24(12) x 100 mm, S&G Biotech) was inserted (B), followed by the deployment of an extended stent graft (SEAL NOVUS Flared Limb Stent Graft Extension 12(14) x 80 mm, S&G Biotech) (C). Post-EVAR angiography confirmed the absence of endoleaks, with contrast filling extending to the right femoral artery (D).

Due to the symptoms and the risk of AAA rupture, elective EVAR was planned. The severe tortuosity of the left common iliac artery presented a significant technical challenge. From left femoral artery access, an Amplatzer™ Vascular Plug II 14/10 (Abbott Laboratories, Abbott Park, IL) was deployed to occlude the left common iliac artery (Figure 2). Subsequently, from right femoral artery access, an EVAR stent graft was placed in the AAA through the right common iliac artery. A SEAL NOVUS Body Stent Graft 24(12) x 100 mm (S&G Biotech) was used for the aorto-uni-iliac component, followed by an extended stent graft SEAL NOVUS Flared Limb Stent Graft Extension 12(14) x 80 mm (S&G Biotech, Yongin, South Korea) (Figure 3). Post-deployment angiography confirmed no endoleak, and arterial flow was established to the right iliac-femoral system (Figure 3).

**Figure 4:**
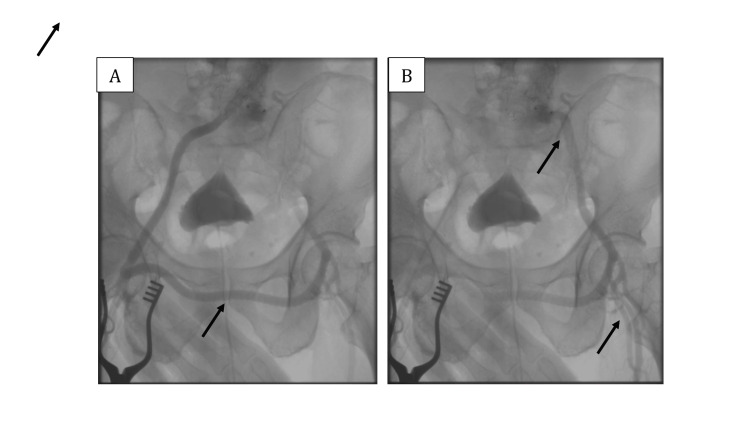
Angiography evaluation post femoral-femoral crossover bypass graft procedure showing the contrast flow through the graft and into the left femoral artery

To restore perfusion to the left lower extremity, a femoral-femoral crossover bypass was performed using a non-kinking vascular graft IMPRA® ePTFE Vascular Graft, Carboflo® Flex Carbon-Lined 8 mm x 40cm (Bard Peripheral Vascular, Inc) with continuous 6-0 Prolene sutures. Completion angiography demonstrated good flow through the graft and into the left femoral artery (Figure 4). The patient had an uneventful recovery, with palpable left lower extremity pulses, no signs of limb ischemia, and a normal ankle-brachial index in both the right and left legs (1.1 and 1.0, respectively).

## Discussion

This case involved a male patient presenting with an infrarenal AAA measuring 63 mm in diameter. According to established clinical ESC guidelines 2024 for the management of peripheral arterial and aortic diseases, the patient's aneurysm size indicated the need for elective intervention [3]. Following a comprehensive analysis of the AAA morphology, and considering the patient's suitable anatomy and a life expectancy exceeding two years, EVAR emerged as the preferred treatment modality [3]. As emphasized by Yamamoto et al. (2015), meticulous patient selection and preoperative planning are critical determinants of successful EVAR outcomes [4]. Thorough patient evaluation encompasses a detailed medical history, including a comprehensive review of comorbidities; a focused physical examination, with particular attention to peripheral pulses and any indications of lower limb ischemia; and advanced imaging studies, notably CT angiography, to precisely delineate aneurysm morphology, assess the iliac arteries, and determine suitability for EVAR. In this case, significant tortuosity of the left common iliac artery precluded a standard EVAR approach. Consequently, a more complex EVAR strategy was adopted, necessitating left common iliac artery occlusion, aorto-uni-iliac stent graft, and the construction of a femoral-femoral crossover bypass graft.

Occlusion of the left common iliac artery in this case was thought necessary to alleviate the risk of endograft fracture and prevent endoleak. The patient's left common iliac artery exhibited severe tortuosity with an angulation exceeding 60 degrees, posing challenges for device insertion and increasing the likelihood of fracture. By occluding the left common iliac artery and utilizing an aorto-uni-iliac stent graft configuration, the risk of fracture due to sharp angulation was minimized, facilitating easier device insertion and reducing the potential for endoleak. The most likely endoleaks for this patient are type I and type III. The landing zone of the EVAR device at both the top and bottom must be securely attached to the blood vessel to avoid type I endoleaks [3]. Device fracture, though a rare complication, may occur in instances of excessive overlap and sharp angulation [5-7]. Such fractures can disrupt the integrity of the endograft and lead to type III endoleak. Fortunately, in the case under discussion, the patient did not develop any of these endoleak complications.

Following the EVAR procedure, the femoral-femoral crossover bypass graft served to restore blood flow to the left lower extremity after left common iliac artery occlusion in this patient. Angiography evaluation showing contrast filling into the left femoral artery through the graft. This successful procedure should be followed by maintaining graft patency and preventing complications such as graft infection, thrombosis, stenosis, and limb ischemia.

Long-term surveillance after EVAR is essential to facilitate the timely detection and management of potential endoleaks. Current guidelines recommend a comprehensive imaging evaluation at 30 days post-intervention, utilizing CCT in conjunction with either duplex ultrasound (DUS) or contrast-enhanced ultrasound (CEUS) to assess procedural success and identify any early complications [3]. While CCT remains the gold standard of endoleak diagnosis, other imaging modalities, including magnetic resonance angiography (MRA), intra-aneurysmal sac pressure measurement, conventional radiography, and digital subtraction angiography (DSA), can also be employed for endoleak detection [8].

## Conclusions

EVAR with left common iliac artery occlusion and a crossover femoral-femoral bypass graft is a viable option for select patients with complex aortic aneurysms and severe tortuosity of the common iliac artery. Of course, the procedure depends on specific anatomical and clinical factors. Collaboration with a multidisciplinary team is essential for optimizing outcomes. Success in these challenging cases hinges significantly on meticulous technique and rigorous patient selection criteria. Consequently, close and consistent long-term follow-up using CCT, DUS, and CEUS is imperative to monitor the repair and ensure durable, positive outcomes.
